# Supplementary material for: Phase-coherent lightwave communications with frequency combs
Source: Nat Commun. 2020 Jan 10;11:201. doi: 10.1038/s41467-019-14010-7 (PMC6954261; doi:10.1038/s41467-019-14010-7)
Supplement: Supplementary file 1 — Supplementary Information [file 41467_2019_14010_MOESM1_ESM.pdf]

Phase-coherent lightwave communications with frequency combs: Supplementary Information

Lars Lundberg, Mikael Mazur, Ali Mirani, Benjamin Foo, Jochen Schröder,
Victor Torres-Company, Magnus Karlsson*, and Peter A. Andrekson.

Photonics Laboratory, Department of Microtechnology and Nanoscience, Chalmers University of Technology, Gothenburg, Sweden

*Corresponding author, email: magnus.karlsson@chalmers.se

Note 1 - Transmission simulations and impairments from delays

This note describes the simulations performed to investigate performance of the master-slave carrier recovery for longer transmission distances than the experiment allowed for. The simulations were done using the split-step Fourier method. It also describes the impairments from delays in the digital processing path and the correlations in the nonlinear phase noise.

The simulated setup was 25×20 GBd 64QAM signals with a spacing of 25 GHz. Only single polarization was simulated. The carriers were assumed to have perfectly correlated phase noise, i.e., the line dependent phase-noise term was neglected. The data on the channels was random and uncorrelated between the channels. The length of the simulated data was 2^{15} symbols with 128 samples per symbol. The step length was chosen depending on the peak power of the signal for each propagated span to ensure 1000 steps per nonlinear length. The span length was 80 km and periodic amplification was used.

After split-step propagation, additional Gaussian noise was added to the signal to simulate transceiver impairments, followed by mixing with an LO with the same linewidth as the carriers. The DSP consisted of frequency shifting, matched filtering, dispersion compensation, adaptive equalization and carrier recovery, either independent or master-slave. The adaptive equalizer was a single-polarization version of the one used for the experimental data, and all DSP parameters were the same as for the experimental data with the exception of the number of taps, which was 11 in the simulations.

The simulations were performed either with a laser linewidth of 50 kHz on the carriers and LO lines, or without added laser phase noise. This way it was possible to study both the effect of dispersive walk-off between the master and the slave as well as differences in nonlinear phase noise. The simulation results can be seen in Supplementary Fig. 1, which includes laser phase noise, and Fig. 2 which assumes ideal, phase-noise free, lasers. By comparing the penalties incurred by master-slave processing in the simulations with and without added laser phase noise, we can see that the penalty caused by the nonlinear phase noise is small compared to the laser phase noise over the distances considered. Without added laser phase noise, the penalty is below or around 0.1 bits/4D-symbol also for a transmission distance of 400 km, while with added laser phase noise, the bandwidth-distance product is limited.

The reason for the increasing penalties with channel separation and transmission distance shown in Figure 1 is the chromatic dispersion in the fiber, which causes a walk-off-induced decorrelation of the phase noise between the different wavelength channels. This will cause the carrier phase noise and the LO phase noise to mix with temporally different parts, incurring phase noise differences in the detected signals [1] that are not possible to compensate electronically. The penalty caused by this effect will depend on the coherence length of the carrier and LO sources, the wavelength separation between the jointly processed channels, the transmitted distance and the dispersion properties of the fiber. Unless optical dispersion compensation is used, this will fundamentally limit

the bandwidth and transmission distance over which joint carrier recovery is reasonable. However, it should be noted that the benefits from master-slave processing is present also for much fewer jointly processed channels than the full simulated bandwidth.

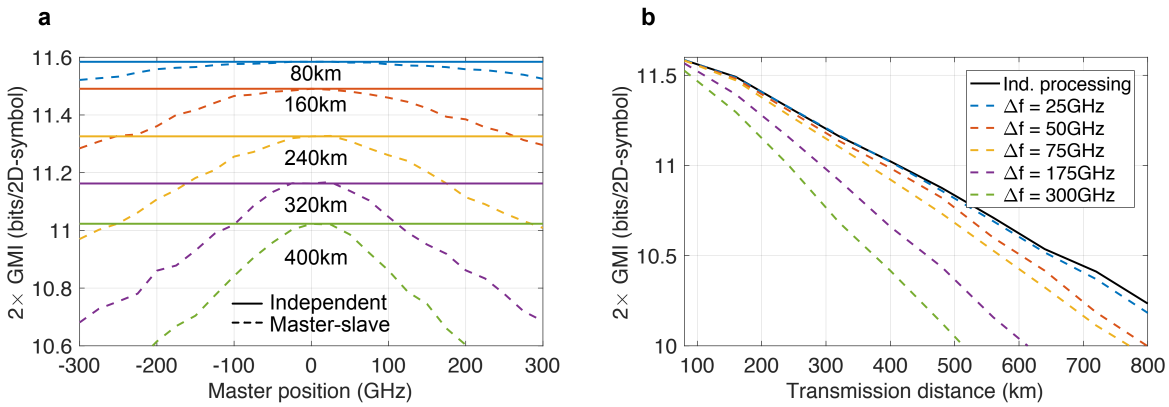

Supplementary Figure 1: Simulation results with laser phase noise The GMI is plotted for the center channel processed either independently (solid) or with master-slave carrier recovery (dashed). (a) GMI as a function of the spectral position of the master channel. (b) GMI as a function of transmission distance. Δf denotes the spectral separation between the master and slave channels.

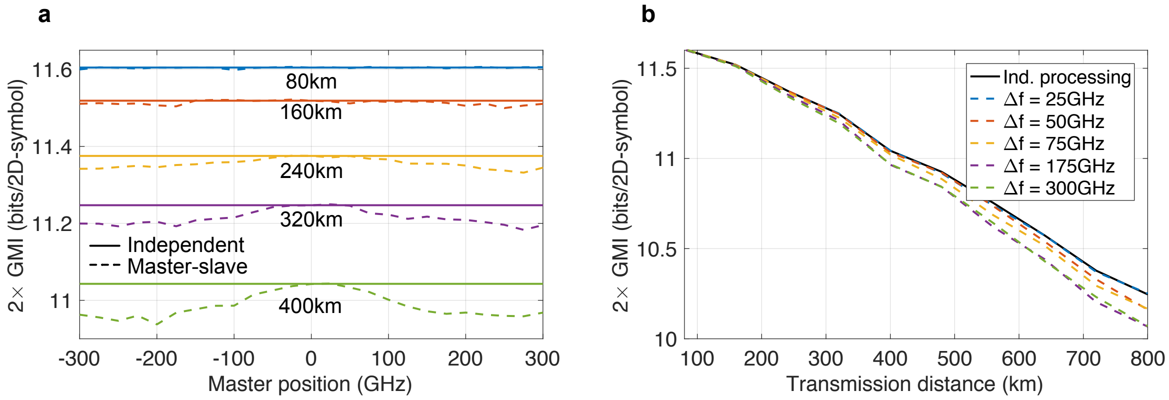

Supplementary Figure 2: Simulation results without laser phase noise The plots are equivalent to Fig. 1, but without added laser phase noise, so that only nonlinear phase noise is taken into account.

In addition to the dispersive walk-off there are two other main limiting factors for joint phase processing, namely the limited comb phase coherence and limitations in the digital electronics. The limited phase coherence of the comb lines is a fundamental property of frequency combs, which manifests as a line dependent phase noise term [2] that increases with the separation between the channels [3]. The significance of this

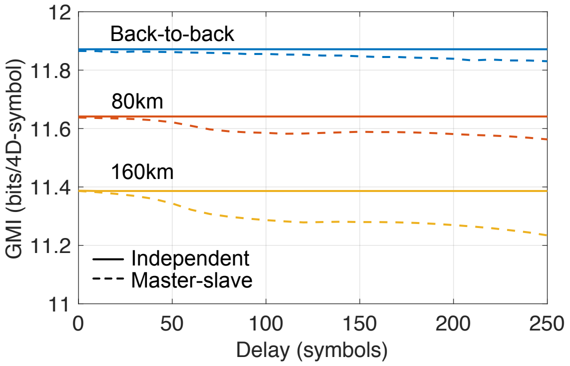

Supplementary Figure 3: Experimental results with digitally inserted delay Comparison of independent and master-slave processing with digitally inserted delay between the master and slave channels. The two channels used are the center channel and one of its neighbors. Optimal launch power is used.

effect depends on the comb generation technique. The electro-optic comb used in this work had a correlation coefficient that was above 99.99% for any line combinations [4].

In addition to the above simulations, we performed an additional experimental check on the tolerable channel skew. With today's technology, practical limitations in the digital electronics are likely to limit the optimal number of jointly processed channels. One key challenge in the implementation of the electronics is maintaining a sufficient time-synchronization between the DSP circuits of many channels. In Figure 3 we in-vestigate this tolerance by introducing an artificial delay digitally between the received master and slave channels. We see that an offset on the order of 50-100 symbols can be accepted, depending on transmission distance.

The limiting factors have in common that they effectively limit the number of channels that can be jointly processed. Therefore, it is important to note that joint processing of already a few channels have benefits. Although our experiments investigate a situation corresponding to several tens of jointly processed channels, a more likely first step in an online DSP implementation would involve only a couple of wavelengths.

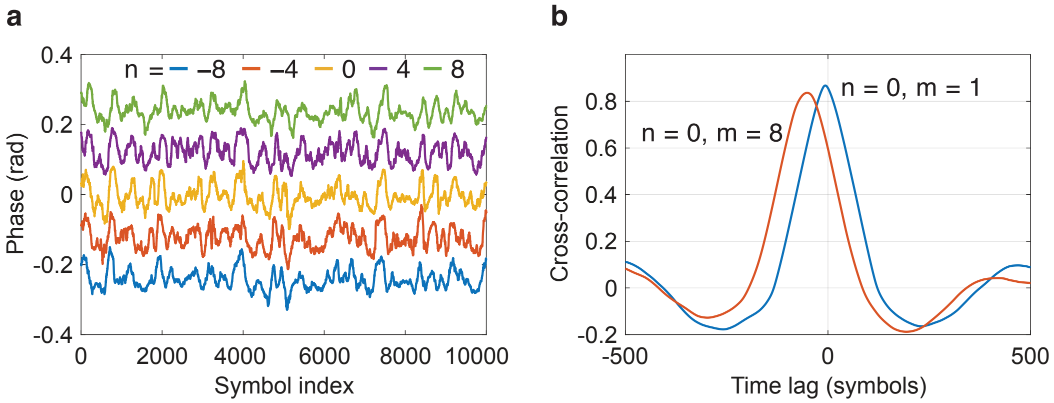

Supplementary Figure 4: Simulated nonlinear phase noise (a) Comparison of the phase extracted from the channels of a setup similar to the one described above1, but with 17 channels in total. The launch power was 1 dBm per channel. No laser phase noise is added. (b) Cross-correlation of the nonlinear phase noise of the center channel with its nearest neighbor and the edge channel. n and m denotes channel indices, where $n = 0$ corresponds to the center channel.

In figure 4a we show, via simulations, how the nonlinear phase noise is indeed correlated among the different channels after 80 km. The phase trace of 5 channels in the plot (channels $\pm 8, \pm 4, 0$) is shown. The cross-correlation function between channels 0,4 and 0,8 is shown in 4b, indicating a shift which is due to the dispersive walk-off. This does indeed verify the potential of the joint processing to mitigate also faster noise fluctuations.

Note 2 - Estimation of transmitter-receiver comb line separation

In this note we investigate how well the difference in comb-line separation between the carrier and LO combs can be estimated from the two received channels.

The frequency spacing is estimated from the data channels through the frequency offset and phase trace estimated using standard independent carrier recovery. The estimated frequency difference is the sum of a coarse frequency difference and a fine frequency difference. The coarse frequency difference is the difference between the frequency offset of the two channels estimated by finding the peak in the 4th power spectrum. The fine frequency difference is then the linear part of the time-dependent phase difference between the two channels. This is estimated by subtracting the phase traces extracted from the two channels using the BPS method. As the phase noise is highly correlated between the channels, the fine frequency difference between them can be estimated by linear regression on the time-dependent phase difference. When the frequency-offset difference between the channels is found, the comb-line frequency-spacing difference can be calculated by dividing with the comb-line index difference of the two channels used. Then we compare this value with the measured spacing difference. The spacing difference is measured by mixing the radio-frequency clocks between the driving the combs.

We evaluated the estimated frequency difference for different frequency spacing between the two channels and for back-to-back, 80 km and 160 km transmission. We used the odd channel set, located at $\pm(25, 75, 125, 175, 225, 275)$ GHz. The results can be seen in Supplementary Figure 5. The frequency difference could be successfully estimated to within a few kHz, which is sufficient to be within the tracking capabilities of the adaptive equalizer. Since the frequency offset difference between the channels is scaled with the channel index difference, a larger spacing between the channels leads to a larger frequency difference to be estimated, which is easier to estimate accurately. This is the reason for the worse accuracy for the cases with smaller distance in channel frequency. The reason for the reduced variation in the estimates at longer distances is not totally clear to us, but is related to the reduced SNR. Based on these results, it can be safely assumed that a joint processing scheme with three or more received channels would have access to information about the comb spacing difference.

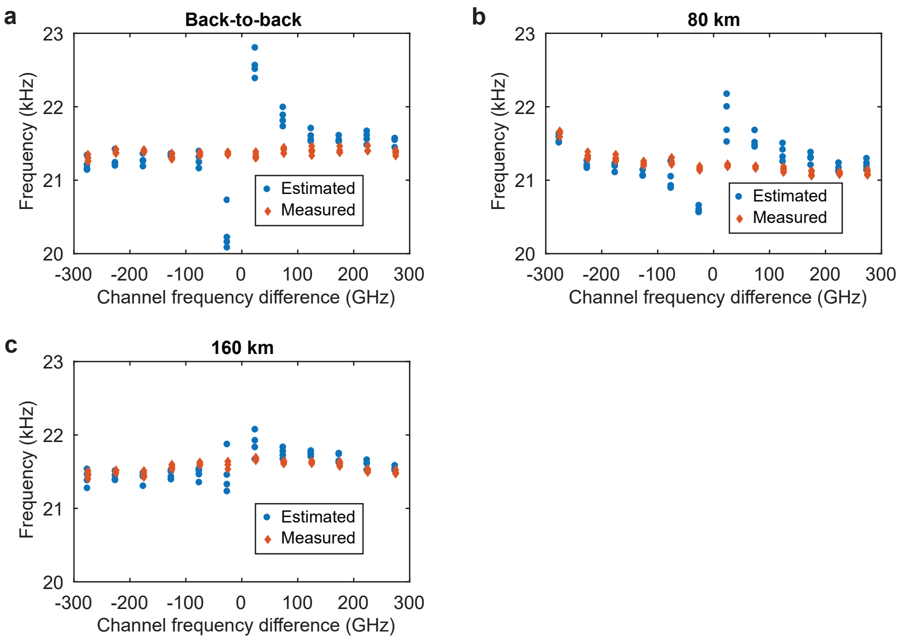

Supplementary Figure 5: Comb-spacing difference estimation Comparison of the spacing difference estimated from the received data or measured from the beatnote of the comb radio-frequency signals. The estimated spacing difference is plotted as a function of the frequency difference between the two channels used for the estimation.

Note 3 - Phase estimation schemes

For the master-slave phase tracking, Figure 6 shows the full signal processing scheme for the phase and frequency recovery, including the tap updates for the polarization trackers.

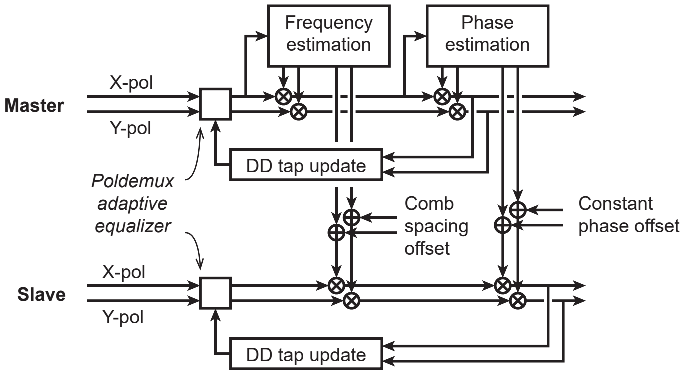

Supplementary Figure 6: Master-slave carrier recovery The frequency and phase is estimated and compensated inside the update loop of a decision directed (DD) adaptive equalizer. A constant frequency and phase offset is added for the slave channel.

For the joint phase estimation scheme, the phases from the two channels and the two polarizations are combined to a joint estimate. In presence of additive noise from amplifiers, an averaging block is needed to improve the signal to noise ratio, under the assumption that the phase reference remains constant under this averaging time. As shown in Figure 7, the joint estimation averages the same amount of symbols under a shorter time, which leads to better tracking performance for rapidly varying phases without sacrificing SNR tolerance. Figure 8 shows the combined frequency and phase estimate in the joint scheme.

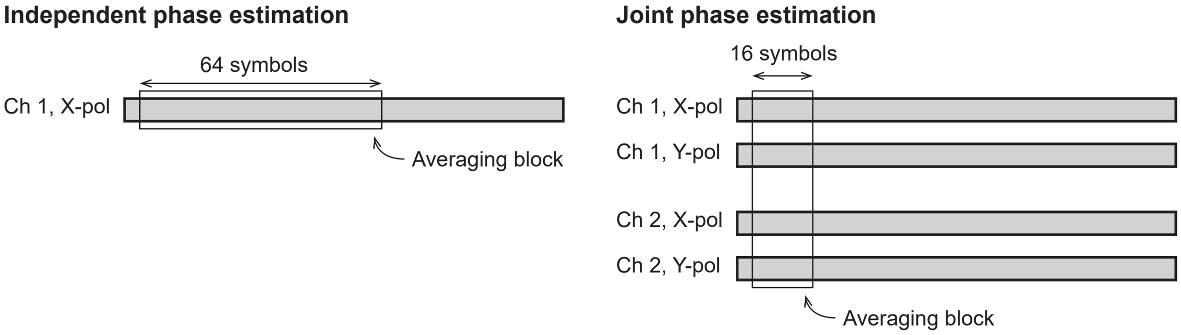

Supplementary Figure 7: Illustration of averaging blocks for independent and joint phase estimation. Comparison of independent and joint phase estimation with the same tolerance to additive noise. In the joint estimation case, the tracking speed is improved.

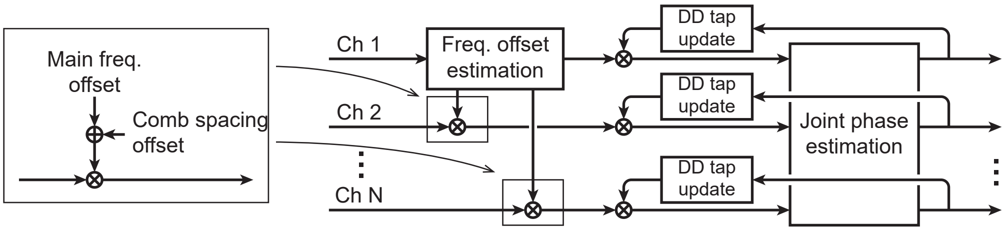

Supplementary Figure 8: Joint phase estimation. Frequency offset compensation is done in a master-slave fashion. One-tap decision directed (DD) equalizers are used to compensate phase differences of channels.

Note 4 - Experimental setup details

A detailed experimental setup is shown in Figure 9 for the transmitter and receiver. Two independent data streams were used to modulate the even and odd channels. The receiver use two synchronized real-time oscilloscopes to detect the desired channels in parallel.

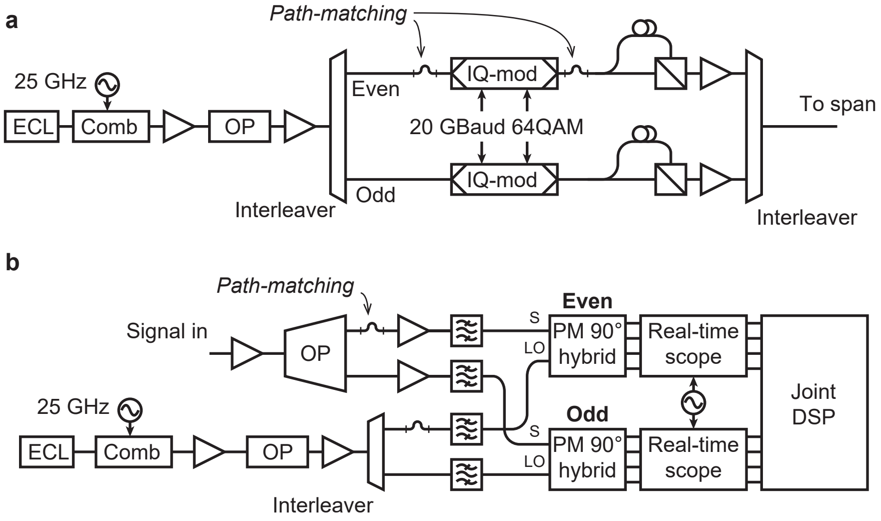

Supplementary Figure 9: Experimental setup (**a**) Transmitter. The lines of an electro-optic frequency comb are modulated with data. (**b**) Receiver. Two channels can be received simultaneously. ECL: External cavity laser, OP: Optical processor, IQ: In-phase quadrature, QAM: Quadrature amplitude modulation: S: Signal, LO: Local oscillator, PM: Polarization multiplexed, DSP: Digital signal processing.

The combs used were two conventional electro-optic combs consisting of a phase modulator in cascade with an amplitude modulator, similar to the combs described in [5]. The transmitter comb was driven by a frequency-multiplied stabilized RF source, and the LO comb was driven by a 25 GHz RF source whose phase noise spectrum is shown in Figure 10.

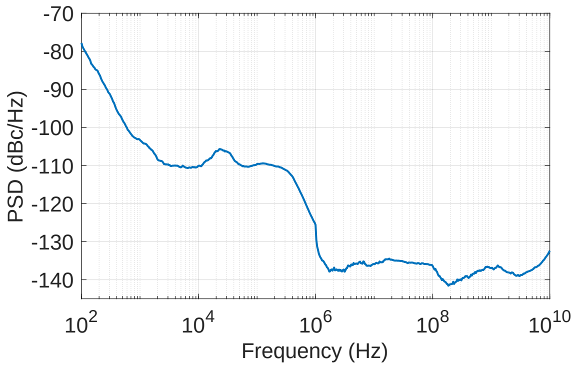

Supplementary Figure 10: Phase-noise spectrum of comb oscillator The phase-noise spectrum of one of the oscillators used to drive the combs used in the experiments.

Supplementary References

[1] Lorences-Riesgo, A., Eriksson, T. A., Fülöp, A., Andrekson, P. A. & Karlsson, M. Frequency-comb regeneration for self-homodyne superchannels. *J. Lightw. Technol.* **34**, 1800–1806 (2016).

[2] Carlson, D. R. *et al.* Ultrafast electro-optic light with subcycle control. *Science* **361**, 1358–1363 (2018).

[3] Lundberg, L. *et al.* Frequency Comb-Based WDM Transmission Systems Enabling Joint Signal Processing. *Appl. Sci.* **8**, 718 (2018).

[4] Lundberg, L. *et al.* Phase Correlation Between Lines of Electro-Optical Frequency Combs. In *Conference on Lasers and Electro-Optics*, JW2A.149 (2018).

[5] Metcalf, A. J., Torres-Company, V., Leaird, D. E. & Weiner, A. M. High-Power Broadly Tunable Electrooptic Frequency Comb Generator. *IEEE J. Sel. Top. Quantum Electron.* **19**, 231–236 (2013).
